# Do fatty acids affect fetal programming?

**DOI:** 10.1186/s41043-015-0018-9

**Published:** 2015-08-13

**Authors:** Seray Kabaran, H. Tanju Besler

**Affiliations:** 1 Department of Nutrition and Dietetics, Faculty of Health Sciences, Eastern Mediterranean University, Famagusta, T.R. North Cyprus via Mersin 10 Turkey; 2 Department of Nutrition and Dietetics, Faculty of Health Sciences, Hacettepe University, Samanpazarı/Ankara, Turkey

**Keywords:** Fatty acids, Fetal programming, Maternal nutrition

## Abstract

**Background:**

In this study discussed the primary and regulatory roles of fatty acids, and investigated the affects of fatty acids on metabolic programming.

**Methods:**

Review of the literature was carried out on three electronic databases to assess the roles of fatty acids in metabolic programming. All abstracts and full-text articles were examined, and the most relevant articles were selected for screening and inclusion in this review.

**Results:**

The mother’s nutritional environment during fetal period has important effects on long term health. Fatty acids play a primary role in growth and development. Alterations in fatty acid intake in the fetal period may increase the risk of obesity and metabolic disorders in later life. Maternal fatty acid intakes during pregnancy and lactation are passed to the fetus and the newborn via the placenta and breast milk, respectively. Imbalances in fatty acid intake during the fetal period change the fatty acid composition of membrane phospholipids, which can cause structural and functional problems in cells. Additionally, the metabolic and neuroendocrine environments of the fetus and the newborn play key roles in the regulation of energy balance.

**Conclusions:**

Imbalances in fatty acid intake during pregnancy and lactation may result in permanent changes in appetite control, neuroendocrine function and energy metabolism in the fetus, leading to metabolic programming. Further studies are needed to determine the role of fatty acid intake in metabolic programming.

## Background

Fatty acids take part in membrane phospholipids, and play important roles in prenatal growth and development. Fatty acids demonstrate their roles in cognitive and behavioral development, and energy metabolism [1, 2]. Maternal nutritional state affects fetal fatty acid supply to the fetus. Fatty acids pass through the placenta during pregnancy, and they are present in breast milk to fulfill their roles in postnatal development [3, 4].

Changes in the metabolic environment, due to insufficient or excessive maternal nutrient intake, can cause short and long-term implications for cell structure and function [2]. Therefore, imbalances in fatty acid intake during fetal development cause structural and functional changes in metabolism [5].

In the fetal period, during organ development, changes in the metabolic environment can lead to the development of chronic diseases in later life [6]. During pregnancy, conditions like maternal obesity, overeating, or malnutrition increase the risk of a metabolic disorder in the fetus [7, 8]. Similarly, overeating during the postnatal stage also increases the newborn’s risk of adulthood obesity [7, 9]. Epidemiological and experimental studies have shown that food substrates supplied to the fetus during pregnancy and to the newborn immediately after birth, can have long term health effects on the development of metabolic diseases, including cardiovascular diseases, type 2 diabetes, hypertension, and obesity [10–15].

One of the mechanisms associated with increasing the risk of obesity is described as metabolic programming, due to the consumption of excessive food during the fetal period and after birth. Studies have shown that excessive food intake by the mother during pregnancy can lead to changes in the fetus’s appetite balance by causing permanent changes in the central nervous system, and this causes hyperphagia after birth [16–19].

Appetite control mechanisms in the hypothalamus have an important effect on the development of chronic diseases. The hypothalamus begins developing in an early stage of pregnancy, and it continues to develop in postnatal life. During growth and development processes, development associated with the hypothalamus is important, and changes in hormonal balance and nutrition status cause changes in the development and function of the hypothalamus [6].

Fatty acids consumed for nutrition have vital functions on energy metabolism and energy storage; they take part in cell enlargement, promotion of cell functions, coordination of intra- and extracellular communications, and regulation of genes that supply energy substrates and control cellular responses to the metabolic environment [20–22]. Therefore, this review will discuss the primary and regulatory roles of fatty acids, and it will explore the involvement of fatty acids in metabolic programming.

## Methods

### Search strategy and selection of studies

To identify eligible studies for this rewiev, a computerized search was performed for all publications available up to March 2013 through PubMed, Web of Science, and EMBASE databases using the following key words: ‘dietary fatty acids’, ‘dietary lipids’, ‘omega-3 fatty acids’, ‘maternal high fat diet’, ‘maternal fat intake’, ‘maternal obesity’, ‘fetal programming’, metabolic programming’, ‘fetal origins of obesity’, ‘early life nutrition’, ‘maternal nutrition’, ‘hypothalamic programming’, ‘developmental programming’, ‘placental lipid transfer’, ‘maternal fatty acid transfer’ and ‘prenatal fatty acid status.’ Reference lists from identified articles and relevant reviews were examined for studies not indexed in the mentioned electronic databases. All abstracts and full-text articles were examined, and the most relevant articles were selected for screening and inclusion in this review. The search was limited to English literature.

## Results

### Fatty acids and growth and development

Long-chain, polyunsaturated fatty acids (LC-PUFA) are important compounds in cell membranes of the central nervous system. Docosahexaenoic acid (DHA), which is found in the brain and retinal membranes, regulates membrane permeability, and improves receptor differentiation. DHA levels also have an effect on enzyme activity [23]. Additionally, DHA has important functions in immune processes, like lymphocyte proliferation, natural killer cell activity, and cytokine production. In addition, DHA and other LC-PUFAs regulate inflammatory and immune responses [24].

The reported effects of omega-3 (n-3) fatty acids on birth weight have turned attention to their effects on growth and development. It was found that low birth weight (52–57 g below average) was associated with the maternal low levels of n-3 fatty acids and dihomo-gamma-linoleic acid (a precursor of arachidonic acid [AA]), but high levels of omega-6 (n-6) fatty acids and trans fatty acids (elaidic acid). Those results indicated that imbalanced maternal fatty acid intakes were associated with impairments in fetal growth and development [25].

DHA is able to pass from mother to fetus through the placenta. Due to the lack of desaturase activity and the low level of fetal enzyme activity in the placenta, the fetus requires placental transfer of LC-PUFAs. Fatty acid transporters, binding proteins, and lipolytic enzymes are responsible for DHA transfer through the placenta to the fetus [23]. In particular, during the last trimester of the pregnancy, LC-PUFAs are required for rapid brain development; thus, optimum development cannot be achieved in premature births [1]. Observational studies have shown that mothers that consumed low amounts of fish during pregnancy had children with higher risks of cognitive and behavioral problems [26, 27]. Similarly, low blood DHA levels in newborns have been associated with low visual and neural maturity [28].

A minimum intake of 160 mg DHA per day increased newborn and maternal DHA levels, and positively affected the development of the central nervous system [2]. In a study conducted with rats, it was determined that supplying about 2.5 % of the energy from n-3 fatty acids (compared to 0.6-1.2 %) positively affected brain development [29]. In addition, a different study showed that, during pregnancy and lactation in rats, both excessive (7 % of the energy) and insufficient (0 %) n-3 intakes resulted in growth deficiencies [30]. Moreover, it was shown that increasing peroxisome proliferator-activated receptor-gamma by consuming n-6/n-3 PUFAs in a ratio of approximately 1-2:1 would be beneficial to brain development [29].

Intrauterine growth retardation results in a low birth weight. A low birth weight is known to have important effects on a child’s growth, development, and health status in later life. Newborns with low birth weights run increased risks of morbidity and mortality in the short term [31] and increased risks of metabolic and cardiovascular diseases in the long term [11–14]. In particular, due to the important effects of fatty acids on fetal growth and development, maternal intake of fatty acids is significantly important for maintaining fetal health [2, 5]. Changes in the metabolic environment, due to insufficient or excessive maternal nutrient intake, can cause cell division, cell differentiation, and programming of cellular responses in the offspring [2].

Fatty acids have important functions in membrane phospholipids. Additionally, fatty acids play complex roles in the formation of transcription factors and receptors that regulate gene expression. Furthermore, fatty acids provide extra- and intra-cellular communication and serve as a precursor to eicosanoids. Therefore, fatty acids play important roles in growth and development. Imbalances in fatty acid intake during fetal development alter fatty acid levels in membrane phospholipids and stored triglycerides, therefore disrupt the cellular environment and cause structural and functional programming [5].

### Maternal fatty acid intake and fatty acid transfer to the fetus

The composition of fats consumed for maternal nutrition affects the properties of the fatty acids that pass through the placenta or are secreted in the breast milk. Thus, it is important to understand the properties of dietary fats [4].

During pregnancy, metabolic changes occur in lipid metabolism and the circulating levels of triglycerides, cholesterol, fatty acids, and phospholipids. These changes with insulin resistance and estrogen stimulation contribute to hyperlipidemia, hyperphagia, lipogenesis, and increases in fat mass and body weight in the 1st and 2nd trimesters. Maternal fat accumulation also causes changes in fetal metabolism, growth, and development. On the other hand, in the 3rd trimester, catabolic lipase activity arises as a result of an increase in lipolytic activity in adipose tissue and a decrease in adipose tissue lipoprotein lipase activity. These changes in catabolic status result in increases in triglyceride, phospholipid, and cholesterol levels. The increase in cholesterol and free fatty acids results in negative effects on fetal metabolism, and it also alters the synthesis of cell membranes and steroid hormones [31]. Thus, changes in nutrients that pass through the placenta can affect fetal metabolism, growth, and development [32, 33].

Essential fatty acids, triglycerides, and lipoproteins consumed during pregnancy reach the fetus by passing through the placenta via special receptors. Upon transfer to the fetus, they bind to alpha-fetoproteins and enter the fetal liver for the synthesis of triglycerides [32]. As mentioned above, the transfer of PUFA/LC-PUFAs is associated with the amounts of maternal fat and maternal intake of fatty acids; additionally, this transfer is affected by placental function and physiological and biochemical processes that arise throughout pregnancy [33–35].

Fatty acids consumed by the mother affect the fatty acid compositions of lipids in fetal blood and newborn blood; in addition, they affect the developing tissues, and cause differentiation in organs and cells. Due to the fact that excessive energy and fat consumption have effects on chronic diseases, the amounts of total fat, saturated fat, and n-6 fatty acids consumed by the mother during the fetal period are considered among factors that affect fetal programming. It was proposed that the most important risk factor associated with fatty acids is excessive n-6 fatty acid intake and insufficient n-3 fatty acid intake [5].

As a result of excessive maternal n-6 fatty acid intake, reduced amounts of n-3 fatty acids, like oleic acid and DHA, are passed to the fetus. Trans fatty acids, found in hydrogenated vegetable oils, are also a part of modern nutrition, and they affect the developing fetus and the health of the newborn baby [36]. Trans fatty acids cause increases in oxidative stress and inflammatory cytokines, which affect metabolic programming. Therefore, when consuming hydrogenated fatty acids, it should be kept in mind, particularly by pregnant women, that they have negative effects on the development of the fetus [10].

### Fetal metabolic programming

Fetal programming implies that during critical periods of prenatal growth, permanent changes in molecular, cellular, metabolic, neuroendocrine, and physiological systems result from adverse intrauterine conditions. These changes, may have long-term consequences to increase the individual's risk for developing adult life diseases. Epidemiological and animal studies have highlighted the link between alterations in the early nutritional environment and increased risk of obesity and metabolic disorders in later life [11–14].

The effect of the fetal period on chronic adult diseases was explained by Barker and Hales [37], who hypothesized that, of all the environmental factors encountered during the early stages of development, nutritional status, in particular, can increase the risk of adult-onset metabolic and cardiovascular diseases [38].

This hypothesis about the fetal period is based on the thrifty genotype hypothesis set forth by Neel [39] in 1962. According to Neel [39, 40], thrifty genes have the ability to store fat by producing more insulin during period of famine, when nutrients are limited, but this situation increases the risks of insulin resistance and type 2 diabetes. A study by Barker and Hales [37] supported the thrifty phenotype hypothesis, when they determined that the development of important organs during the fetal period changed by adapting to a state of limited nutrients. This change ensured survival in a nutrient-deficient environment during postnatal life. However, when abundant nutrients are encountered after birth, long term harmful effects occur due to the incompatibility between prenatal and postnatal environments [41–43].

An absence of nutrients in the intrauterine environment causes problems, like reduced numbers of nephrons, heart muscle cells, and pancreatic cells. As a result, the functional capacities of these organs are inadequate throughout the life. It is thought that reductions in cell numbers and functional capacities occur as a result of reduced energy density passing through the placenta; thus, they represent adaptations aimed to protect the development of important vital organs, like the brain [9, 11]. Barker and Osmond [44], conducted a study with 16,000 people and determined that low birth weight was associated with increased risks of diseases, like coronary heart disease, hypertension, and type 2 diabetes. Low birth weight results from insufficient nutrition; thus, inside the uterus, vital fetal organs become conditioned for a nutrient-deficient environment. Thus, it is thought that encountering a different environment after birth increases the risks of chronic diseases [41–44].

In addition, epigenetic mechanisms contribute to gene expression throughout development. Epigenetic modifications in regulatory genes and growth-related genes play a significant role in fetal programming. Differences in fetal gene expression inside the uterus can cause epigenetic modifications [45]. Epigenetic modifications that involve gene regulation can cause changes in organ structures, cell numbers, and metabolism. Additionally, epigenetic modifications can involve a number of processes, like apoptosis, that affect growth and development [46, 47]. Moreover, it was noted that endocrine responses involved in prenatal development are affected significantly by nutritional status. During the prenatal stage, many hormone signaling pathways are affected, and tissue sensitivities to a variety of hormones change. Thus, during the development process, permanent changes can occur [48].

In the past, the effect of insufficient maternal nutrition on adulthood chronic diseases was emphasized; however, currently, it is thought that maternal obesity poses a higher risk to the offspring [8, 49]. Maternal obesity, gestational diabetes, and preeclampsia can cause changes in fetal growth and development, similar to those caused by mother-related insufficient nutrition, placenta-related dysfunction, mother-related stress, and other environmental influences (infections, alcohol, tobacco) [9, 49]. Therefore, both low and high birth weights may generate physiological and/or metabolic adaptations in primary vital organs (brain) and other important organs (pancreas, kidneys, muscles, and liver) [49–51].

### Fatty acids and metabolic programming

It is indisputable that excessive energy intake can cause obesity, and both maternal and adolescence obesity are increasingly becoming issues [52, 53]. It was mentioned that metabolic stress factors encountered during both prenatal and postnatal stages can affect health in later life. For example, maternal obesity or diabetes during pregnancy or lactation, low birth weight, and rapid weight gain in preterm babies are factors associated with obesity, glucose intolerance, insulin resistance, dyslipidemia, and high blood pressure. Studies conducted with animals have shown that excessive intakes of fat, saturated fat, and carbohydrates or low intakes of protein and vitamins during pregnancy and lactation can lead to increased risk of metabolic syndrome [10–14].

Fatty acids are affected by maternal nutrition; they pass through the placenta or breast milk and accumulate in the fetus or newborn, respectively [4]. In prenatal and postnatal stages, fatty acids and metabolites play an important role in cell enlargement and differentiation, and they regulate cellular responses between metabolic and neuroendocrine environments [2]. Although fatty acids are mainly found in the phospholipids of cell membranes, they also contribute to the molecular signals that govern appetite and energy metabolism. In addition, fatty acids act as metabolic sensors that participate in the regulation of genes involved in energy oxidation and storage. Therefore, it is thought that insufficient, imbalanced, or excessive fatty acid intake in the early stages of development can also contribute to metabolic programming [5]. In a study that included 150 pregnant women with gestational diabetes, but with controlled blood sugar, a correlation was found between serum free fatty acid levels and fetal fat mass in the 3rd trimester. It was suggested that this condition could cause long term effects on health (e.g., dyslipidemia, hypertension, insulin resistance, and obesity) [54].

Changes in nutrition affect the endocrine system, which, in turn, causes disruptions in appetite mechanisms and energy balance. Inappropriate metabolic and endocrine responses occur in organs like brain, adipose tissue, muscle, liver, and pancreas [14]. Although the mechanisms that give rise to these inappropriate organ responses are not completely understood, it is thought that structural changes in the cells and organs occur due to changes in metabolic and neuroendocrine signaling, gene expression, and epigenetic mechanisms [5, 55]. For example, epigenetic modifications, like DNA methylation and histone modification play key roles in biological processes involved in intrauterine development, including gene expression, chromatin accessibility, and DNA replication. These processes are important in forming, protecting, and transferring phenotypes to the next generation [10, 11]. DNA methylation is affected by factors associated with nutrition. Methylation of some genes is initiated by metabolic and neuroendocrine events. It is thought that those genes are responsible for epigenetic programming, depending on the conditions in nutritional or endocrine environments [11]. It is also thought that maternal fatty acid intake can cause epigenetic modifications [10].

In recent years, studies on metabolic programming have focused on excessive maternal nutrition. These research studies involved women that were overweight or obese during pregnancy. With increases in maternal body weight and triglyceride levels, there is an increase in maternal fatty acid transfer, which affects the body weight, adiposity, inflammatory cytokines, and leptin levels of the newborn infant. All these factors prepare the ground for the development of hyperphagia, insulin resistance, obesity, and type 2 diabetes [8, 49, 56]. Studies conducted with animals showed that high fat or high carbohydrate intakes during pregnancy or lactation caused increased lipid accumulation in muscles, adipose tissue, and liver of the offspring and changes in hepatic gene expression, pancreatic beta cell development, and insulin and leptin secretions [56–61].

In a study conducted with rats during pregnancy and lactation, one group was fed a normal diet and another group was fed a diet with 30 % less energy than normal. The offspring born after insufficient gestational nutrition were then given a hypercaloric diet with 30 % of the energy from fats. In that group, the average daily energy intake per body weight was increased and, when the offspring reached 100 days old, their systolic blood pressure and blood insulin levels were elevated [58]. In a different study conducted with female rats, during the 120 days prior to pregnancy, one group was fed a diet with 59.5 % of the energy from fats (high fat), and another group was fed a diet with 10.9 % of the energy from fats (standard diet). On the 21st day of gestation, compared to the standard diet group, the high fat diet group was 20 % heavier, consumed more energy, and had twice insulin, 34 % more glucose, and 68 % more triglycerides in their blood. In a continuation of the study, aimed at investigating the effects of hypercaloric nutrition, they tested male rats born to the mothers fed a high fat diet. After the lactation period, one group was fed standard nutrition, and another group was fed a high sucrose (67 % carbohydrate, 7 % fat, and 18 % protein) diet. Compared to the standard nutrition group, the group fed a high sucrose diet (after a high fat intake during pregnancy and lactation) exhibited elevations in body weight, plasma triglycerides, free fatty acids, and insulin levels. Hence, this condition caused metabolic problems [59].

In a study hypotesised that high-fat feeding during pregnancy is associated with a programming effect on the liver. It was concluded that maternal high-fat diet feeding during pregnancy programs liver which strongly modulates glucose homeostasis and organ fat accumulation in adult life after exposure to a nutritional insult [61]. High fat or high sugar diets consumed during the perinatal stage can cause permanent changes in metabolism and lead to excessive consumption of appetizing foods. Another study investigated the effects of junk food-type maternal nutrition during pregnancy and lactation stages on 6-week to 3-month old rats. They determined that rats born to mothers fed junk food had higher fat consumptions compared to those born to mothers fed a standard diet [60].

Similar to the amount of total fat intake, the type of fatty acid consumed also has effects on organ development and metabolic programming [62–64]. In a study conducted with rats, a diet containing 18 % fat was fed to pregnant rats for 2 weeks. One group was given coconut oil (saturated fatty acid), another group was given fish oil (unsaturated fatty acid), and a third group was given soy oil (control group). The effects of fatty acids on the health of the newborn were investigated. That study determined that the offspring of mothers fed unsaturated fats had lower birth weights and slower weight gain after birth than offspring from the other groups. The offspring from mothers fed saturated fats exhibited low numbers of pancreatic islets and a rapid, high insulin response on the oral glucose tolerance test. Therefore, it was determined that the type of fatty acids consumed during pregnancy could have effects on the growth, pancreatic development, and glucose metabolism of the newborn [62].

In a study conducted with rats, hydrogenated vegetable oils, palm oil, canola oil, and soy oil were used to determine the effects of different fatty acids on adiposity and blood lipids. The four groups of fatty acid compositions were fed to mothers during pregnancy and to the offspring after birth, for 21 days during and 45 days after the lactation stage. The results showed that the offspring in the palm and hydrogenated vegetable oil groups had higher body weights than those in the canola and soy oil groups. Furthermore, the hydrogenated vegetable oil group exhibited higher triglyceride and total cholesterol levels than those of the palm oil group, and lower HDL cholesterol levels than those of the other 3 groups [64].

It was observed that, as a result of the energy, total fat, and fatty acid intakes of the mother during pregnancy and lactation, metabolic programming could be caused in many organs of the offspring, including adipose tissue, muscle tissue, liver, and pancreas. These changes in main vital organs indicated that the incompatibility between the early stage food substrates and the endocrine environment can cause permanent changes due to programming of hypothalamic signaling, appetite, and energy balance [5]. These studies provided important information about the potential long term effects of a high fat maternal diet or maternal consumption of different types of fatty acids on the metabolic programming of offspring [58–63].

## Discussion

It is claimed that the fetal environment has effects on hypothalamic and brain functions that involve appetite control, energy balance, and obesity [65, 66]. Brain development starts during the development of the embryo and continues after birth [6]. Brain development has effects on critical developmental stages, like cell proliferation, transport, differentiation, axon development, synaptogenesis, and apoptosis. Disruption of hypothalamic development during critical development stages causes serious structural and functional anomalies in the hypothalamus [67].

The hypothalamus combines endocrine, metabolic, and neural signals to form behavioral, anatomic, and endocrine responses, and hence, provides appetite control [6]. Animal experiments have shown that maternal obesity can cause programming of hypothalamic appetite control. High fat nutrition during pregnancy and lactation causes hyperphagia and obesity in the offspring, and it disrupts hypothalamic gene expression [18, 68]. A study conducted with mice showed that differences in prenatal and postnatal nutrition, particularly insufficient prenatal protein intake combined with high fat postnatal intake, caused changes in appetite metabolism due to excessive nutrient intake [69].

Regulation of energy balance and appetite is complex. It involves balancing the important orexigenic neuropeptides, like neuropeptide Y (NPY) and agouti-related peptide (AgRP), and anorexigenic peptides, like proopiomelanocortin (POMC) and cocaine and amphetamine regulating transcript (CART). Additionally, hypothalamic control is affected by hormones secreted from the adipose tissue, pancreas, and intestines [6, 11]. Leptin, secreted by adipose tissues, and insulin, secreted by the pancreas, are among the most important hormones that regulate energy balance. When these hormones penetrate the blood–brain barrier, neural signals in the acute nucleus part of the hypothalamus also take part in regulating energy balance. NPY, AgRP, and POMC are produced by acute neurons. Leptin and insulin directly target AgRP, NPY, and POMC production; they activate POMC neurons and block NPY neurons. These actions cause a reduction in nutrient intake and an increase in energy consumption [70].

During the early stages of development, changes in the signals from leptin and insulin alter the development of neuro-regulatory signals that control eating behavior. It is thought that these changes may be responsible for hyperphagia or excessive weight gain in later life. It was shown that a maternal high fat diet caused changes in hypothalamic peptides, like POMC and AgRP, in the fetal brain [71, 72]. In an experimental study, it was determined that high fat maternal nutrition caused leptin resistance in the offspring. The development of leptin resistance, hyperphagia, and increased adiposity cause increases in the risk of adult obesity [18]. Additionally, maternal obesity and increased food intake disrupt glucose metabolism, and raise insulin resistance in the offspring; this increases the risks of adiposity and obesity [8, 73].

The melanocortin system in the hypothalamus also plays a vital role in regulating energy balance and body weight. Melanocortins are anorexic neuropeptides produced from the POMC gene. AgRP, on the other hand, works in opposition to the melanocortin system and stimulates appetite [72]. It is known that mutations in melanocortin receptors cause hyperphagia and obesity in mice and humans [74, 75]. High fat maternal diet and maternal obesity cause fetal and neonatal liver lipotoxicity [8, 76]. Moreover, high fat maternal nutrition increases inflammatory cytokines in offspring [72]. Both high fat nutrition and elevated inflammatory cytokines affect the melanocortin system [72, 77].

The hypothalamic melanocortin system, which controls appetite and energy consumption, develops during the last trimester. Maternal nutrition and the health of the mother have important effects on the development of the melanocortin system. A study was conducted with female monkeys to investigate the effects of high fat maternal nutrition during pregnancy on the development of the melanocortin system in offspring. The monkeys were fed a high fat (35 % fat, peanut oil) or controlled (13 % fat) nutrition diet for up to 4 years; during that time they became pregnant, and the fetuses were examined in the 3rd trimester. One group of monkeys on the high fat diet were switched to the controlled diet in the 5th year; during that time they became pregnant. Compared to fetuses from the controlled diet group, those from the high fat group showed higher POMC mRNA expression and lower AgRP mRNA expression. Additionally, they found increased levels of proinflammatory cytokines including IL-1β and IL-1 type 1 receptors in the hypothalamus. The offspring of the monkeys that switched to a controlled diet during the 5^th^ year had normal melanocortin levels. Independent of maternal obesity and diabetes, high fat nutrition during pregnancy can cause activation of proinflammatory cytokines, which can change the development of the melanocortin system. When fetal POMC system anomalies continue during the postnatal stage, a variety of body systems may be affected, including body weight maintenance, stress responses, and cardiovascular functions. Moreover, a high fat diet causes the offspring to gain excessive weight at an early stage. These anomalies can be prevented in the offspring by sufficient, balanced nutrition in pregnant women, regardless of obesity or serious insulin resistance [72].

Dietary lipids affect the fatty acids present in neuronal membranes, some neurotransmitters, and lipid-like signaling molecules. Thus, changes in programming can affect adipose tissue mass, compositions of lipids, and metabolism [5]. A study conducted recently on rats investigated fat mass accumulation over 4 generations when the diet included 35 % of the energy from fats (18 % linoleic and 0.6 % alpha-linoleic, 28:1). They found that, although food consumption remained the same in each generation, adipose tissue mass, hyperplasia, and hypertrophy gradually increased over 4 generations. Moreover, it was observed that, over the 4 generations, adipokine levels changed, adipose tissue gene expression changed, and hyperinsulinemia developed. Considering all these changes, it is reasonable to hypothesize that the modern increase in obesity may be associated with excessive linoleic acid intake [78].

In another study, the adiposity status of 3 year-old children was compared with fatty acid intake during the 2nd trimester of pregnancy. They measured total skin fold thickness of the triceps and subscapula. Those authors could not find a significant relationship between the maternal eicosapentaenoic acid (EPA) + DHA plasma concentration during pregnancy and the adiposity of their children. However, they found that increases in the n-6/n-3 ratio in the maternal diet were related to increases in the adiposity of the children. Therefore, it was determined that insufficient n-3 intake during pregnancy may be a risk factor for childhood obesity [79]. In addition, a recent study determined that a higher intake of n-6 fatty acids compared to n-3 fatty acids was associated with increased adipogenesis [78]. Another study showed that a high n-6/n-3 intake during pregnancy caused an increase in the number of fat cells of the developing fetus; consequently, the n-6/n-3 ratio is considered a risk factor for childhood obesity [80]. It was determined that DHA prevented adipocyte differentiation, initiated apoptosis in preadipocytes, and increased lipolysis in adipocytes; these activities were expected to create a protective effect against obesity [81]. In a different study, it was stated that, particularly in perinatal stages, n-3 fatty acids can play a role in regulating appetite signals, and they can affect the normal development of food intake mechanisms [82]. A systematic evaluation of the results from animal studies on how maternal n-3 LC-PUFA supplementation affected the body mass of offspring emphasized that more research is needed on this subject [83]. Although, several studies have shown that n-3 fatty acid intake during pregnancy has a protective effect against obesity, more studies are needed to confirm the results.

When the effects of fatty acids on body functions are considered from all different aspects, it can be claimed that they have an effect on metabolic programming. Saturated fats, n-3 fatty acids, and n-6 fatty acids have different effects on lipid metabolism [84]. In the liver, fatty acids regulate the expression of key lipogenic, lipolytic and glycolytic genes [21]. Fatty acids or metabolites affect transcription factors that regulate the expression of enzymes that regulate gluconeogenesis, glycolysis, fatty acid synthesis, and fatty acid oxidation [84]. Micronutrients, like folic acid and vitamin B12, also have effects on fatty acid metabolism, and these are considered nutritional factors that can influence metabolic programming [85].

Based on all these metabolic processes, it is clear that fatty acids, particularly n-6 and n-3 fatty acids, can have effects on fetal programming. The effects of these fatty acids on fetal programming are transmitted in their roles as bioactive compounds, in cell differentiation, in metabolic signal regulation, in energy balance mechanisms, and their potential functions in other physiological processes.

## Conclusion

This review discussed a variety of hypotheses on metabolic programming. We emphasized the effects of maternal fatty acid intake on fetal development by detailing the primary functions of fatty acids on growth and development. The facts that fatty acids are a main energy source, play key roles in metabolic processes, and have important functions on appetite and energy balance, indicate that they can affect the metabolic programming of obesity and other chronic diseases.

Currently, coincident increases in the frequency of metabolic diseases and consumption of n-6 fatty acids have called attention to the point that fatty acids may have effects on metabolic diseases. Although insufficient evidence exists to support this point, studies with animals have suggested that fatty acids may have important effects on human health. Fatty acids contribute to complex mechanisms, affect multiple tissues, and take part in multiple metabolic processes.

The Institute of Medicine (IOM) recommends the consumption of 200-300 mg/day DHA from seafood during pregnancy and lactation. To meet these recommendations, an average of 340 g/week of fish should be consumed. Swordfish, shark, and large mackerel should be avoided, because they may contain methyl-mercury [86].

Excessive or insufficient fatty acid intake during the prenatal and postnatal stages cause metabolic and endocrine adaptations that affect cell division, cell differentiation, gene expression, and epigenetic modifications. All these changes can, through developmental programming, cause permanent changes in body composition, adipose tissue, vital organs (like brain and liver), and metabolic and neuroendocrine systems. The studies discussed here were focused on excessive and high fat maternal nutrition, and the general properties of fatty acids were put into the context of their potential effects on energy balance. In future, long term observational studies are needed to determine the effects of fatty acids on fetal programming.
